# One size does not fit all: Evaluating disparities in lung cancer screening eligibility amongst the Hispanic population

**DOI:** 10.3389/fonc.2022.995408

**Published:** 2022-11-01

**Authors:** Coral Olazagasti, Matthew Ehrlich, Nagashree Seetharamu

**Affiliations:** ^1^ Division of Medical Oncology at Sylvester Comprehensive Cancer Center, University of Miami, Miami, FL, United States; ^2^ New York Presbyterian/Columbia University Medical Center, New York, NY, United States; ^3^ Donald and Barbara Zucker School of Medicine at Hofstra/Northwell Health, New Hyde Park, NY, United States

**Keywords:** lung cancer, screening, tobacco, early detection, disparities

## Abstract

Lung cancer (LC) is the leading cause of cancer death among Hispanic men. We assessed the tendencies for screening eligibility amongst Hispanic prior to LC diagnosis according to the NCCN and The USPSTF guidelines available at the time of diagnosis. We conducted an observational study in patients diagnosed with LC from 2016 to 2019. Charts were reviewed to assess their screening eligibility prior to LC. The chi-square test was used to examine the association between race and ethnicity with each screening criteria. A total of 530 subjects were reviewed, of which 432 were included in the analysis. One hundred fifty-three and 245 subjects were ineligible for screening under NCCN and USPSTF criteria prior to their LC diagnosis. Twenty-eight of the subjects who did not fulfill NCCN criteria identified as AA and 12 as Hispanics. Forty and 20 of the USPSTF screening ineligible subjects identified as AA and Hispanics. There was a significant association between screening eligibility criteria in Hispanics, with 52% Hispanic subjects meeting NCCN criteria compared to only 20% who met USPSTF (p=0.0184). There was also a significant association between ethnicity and USPSTF eligibility criteria (p=0.0166), as 80% of Hispanic subjects were screening ineligible under USPSTF criteria compared to 56% of non-Hispanic or other. In our study, Hispanics had significantly lower tendencies of meeting the USPSTF LC screening eligibility criteria than non-Hispanics or other. Interestingly, a proportionally higher number of Hispanics who were ineligible under USPSTF criteria met NCCN criteria. These findings suggest that leniency in the screening criteria can possibly lead to earlier detection of LC in high-risk individuals. Recently, USPSTF has modified their criteria which may benefit more of these individuals. To improve rates of screening and overall mortality of minorities, organizations should continue to re-evaluate and liberalize their screening guidelines.

## Introduction

Lung cancer is the leading cause of cancer-related death worldwide, accounting for almost 25% of all cancer deaths (1). The five-year overall survival for lung cancer remains less than 20% (2). Its incidence and mortality rate are even more pronounced within certain subgroups, where racial disparities are particularly predominant (1). African Americans have the highest rates of LC mortality in the United States and the second-highest LC incidence. African Americans are also more likely to develop LC at an earlier age and present with advanced-stage disease (3). While the incidence is not as high amongst Hispanics, LC is the leading cause of mortality in Hispanic men and the second-leading cause of cancer mortality in Hispanic women. The survival rates for Hispanics are lower than those for Non-Hispanic Whites (NHW), mainly due to lower rates of early diagnosis and screening. Compared to non-Hispanic Whites, Hispanics have higher rates of being diagnosed at advanced stages of lung cancer, discarding their candidacy for surgical resection and curative intent (4).

In the last two decades, two landmark prospective randomized-controlled studies – the National Lung Screening Trial (NLST) and the Dutch–Belgian Lung Cancer Screening Trial (NELSON) – demonstrated that screening with annual low-dose computed tomography (LDCT) reduces lung cancer mortality among high-risk individuals (5, 6). On the other hand, the Multicentric Italian Lung Detection (MILD) trial assessed the mortality rates from lung cancer by comparing annual and biannual screening with LDCT. However, the study revealed a similar overall mortality between both arms (7). Organizations such as the National Comprehensive Cancer Network (NCCN) and U.S. Preventative Services Task Force (USPSTF) extrapolated the findings of the aforementioned trials to create lung cancer screening guidelines in 2011 and 2013, respectively, recommending annual LDCT among high-risk adults. The NCCN11 classified high-risk patients as those ages 55-74 with ≥ 30 pack-year history of smoking with <15 years since smoking cessation; or ≥20 pack-year history of smoking, and additional risk factors that increase the risk of lung cancer to >1.3%, which include: family history of lung cancer, personal history of other malignancy, history of COPD or pulmonary fibrosis, radon exposure, occupational exposure, and/or second hand-smoking exposure (8). The USPSTF13, on the other hand, recommended annual screening for lung cancer in adults aged 55-80 years with ≥ 30 pack-year smoking history, current smokers or those that had quit within 15 years (8, 9) Unfortunately, however, subsequent analysis found that certain minority populations were underrepresented in these trials. The participants in the NLST were predominantly white (95%), and only 1.8% of the participants were Hispanics – likely leading to some of the racial and ethnic disparities in lung cancer screening that we see today (5).

Many studies have published data citing lower rates of screening eligibility and low dose CT implementation in the African American population. There was a secondary analysis of the NLST that demonstrated an even greater reduction in lung cancer and all-cause mortality in African Americans compared to White, despite low participation (4.4% black vs 90.9% white) (10). Additionally, African Americans have been shown to have lower lung cancer screening eligibility rates despite having greater incidences of lung cancer (11, 12). Regretfully, the efforts have focused mainly on this minority group and limited data exists understanding the eligibility patterns and screening uptake in the Hispanic population. In efforts to understand the patterns and the factors that contribute to these inequities, we conducted a secondary analysis of an observational study (13) to evaluate the tendencies for screening eligibility among the Hispanics population prior to their lung cancer diagnosis. To our knowledge, no previous studies have been published that highlight a potential for missed opportunities in high-risk, underserved groups such as the Hispanic populations that are eventually diagnosed with lung cancer.

## Methods

### Study description

We conducted a secondary analysis from a single-center observational study in an outpatient Academic Center that originally sought to retrospectively assess the rates of lung cancer screening uptake in subjects with lung cancer, prior to their diagnosis The study protocol was reviewed by institutional review board and the need for approval was waived by Northwell Health Institutional Review Board (IRB #190580)We reviewed the charts of consecutive patients with an established diagnosis of LC at the Northwell Health Cancer Institute between 2016 and 2019. Charts were reviewed for demographics, detailed smoking history at the time or prior to screening, family history, history of previous malignancy, radon exposure, occupational exposure and/or second hand-smoking exposure to assess lung cancer screening eligibility prior to the diagnosis of lung cancer.

In this *ad-hoc* analysis, we aimed to assess the patterns of lung cancer screening eligibility according to NCCN11 and/or USPSTF13 criteria in patients prior to their diagnosis of cancer. Our primary endpoint was to compare the NCCN11 and USPSTF13 rates of screening eligibility according to race and ethnicity. We also sought to understand potential disparities in LDCT uptake according to sex, race, and ethnicity.

### Statistical methods

Subjects were considered to have fulfilled LC screening criteria if they met eligibility according to NCCN11 and/or USPSTF13 LC screening guidelines. Those who did not meet either of the criteria were considered screening ineligible. Subjects who had missing information that was required for determining eligibility for either or both criteria were not categorized and excluded from analysis. All analyses were carried out separately for each screening criteria (NCCN11 and USPSTF13). The association between each categorical demographic and clinical factor and referred for screening (yes/no) was examined using the chi-square test or Fisher’s exact test. The association between screening eligibility with race and ethnicity was examined using the chi- square.

## Results

Charts of 530 subjects were reviewed, of whom 432 were current or former smokers and 98 had no history of smoking. Baseline characteristics are shown in **Table 1**.

**Table 1:** Baseline characteristics and characteristics at diagnosis.

	SmokersN (%)	Never SmokersN (%)
**Frequency**	432 (82.0)	98 (18.0)
**Baseline Characteristics**		
Gender*		
** Male**	231 (55.1)	27 (27.6)
** Female**	188 (44.9)	71 (72.4)
Race**		
** African American**	65 (15.1)	18 (18.4)
** White**	295 (68.5)	40 (40.8)
** Asian**	26 (6.0)	29 (29.6)
** Other**	45 (10.4)	11 (11.2)
Ethnicity**		
** Hispanic**	25 (5.8)	6 (6.1)
** Non-Hispanic**	392 (91.0)	87 (88.8)
** Other**	14 (3.2)	5 (5.1)
Primary Language**		
** English**	399 (92.6)	78 (79.6)
** Other**	32 (7.4)	20 (20.4)

*Missing data for 13 subjects with smoking history.

**Missing data for 1 subject with smoking history.

Out of the patients with a smoking history, 55% were male and 45% female. White was the most prevalent race, with 68.5% participants self-identifying as White, whereas 15.1%, 10.4% and 6.0% self-identified as AA, other, and Asian. In terms of ethnicity, up to 91% of participants identified as Non-Hispanic, 5.8% identified as Hispanic, and 3.2% as other. English was the primary language for 93% of participants.

### Screening eligibility

**Table 2** depicts the Screening criteria eligibility per race and ethnicity. A total of 245 of participants with a history of smoking were ineligible for lung cancer screening according to NCCN11 prior to their lung cancer diagnosis. When assessing the relationship between NCCN11 eligibility and race, 43% of the self-identified African American subjects and 34% of Whites, Asian, or other subjects were ineligible for lung cancer screening per NCCN11 criteria (p=0.206). When comparing NCCN11 eligibility and ethnicity, 48% of the self-identified Hispanic subjects and 35% of non-Hispanicsor others did not fulfill NCCN11 eligibility criteria (p=0.201).

**Table 2:** Screening criteria eligibility per race and ethnicity.

	NCCN Eligible		p-value	UPSSTF Eligible		p-value
	Yes(%)	No(%)		Yes(%)	No(%)	
**Race** African American White, Asian, other	56.9 65.6	43.1 34.4	**0.206**	38.5 43.8	61.5 56.2	**0.496**
**Ethnicity** Hispanic Non-Hispanic or other	52.0 65.0	48.0 35.0	**0.201**	20.0 44.4	80.0 55.6	**0.017**

Out of the patients with a smoking history, 153 did not fulfill USPSTF13 eligibility criteria prior to their diagnosis of lung cancer. When assessing the relationship between USPSTF13 eligibility and race, 62% of the self-identified African American subjects and 56% of Whites, Asian, or other did not fulfill USPSTF13 eligibility criteria (p=0.496). When comparing USPSTF13 eligibility and ethnicity, 80% of the self-identified Hispanic subjects were ineligible for screening, compared to 56% of non-Hispanics or others (p=0.017).

There was a significant association between screening eligibility criteria (NCCN11 and USPSTF13) in Hispanics, where 52% of HispanicX subjects were eligible according to NCCN11 criteria compared to only 20% Hispanics who fulfilled USPSTF13 eligibility criteria (chi-square 5.555; p=0.0184).

### Screening uptake

**Figure 1:**
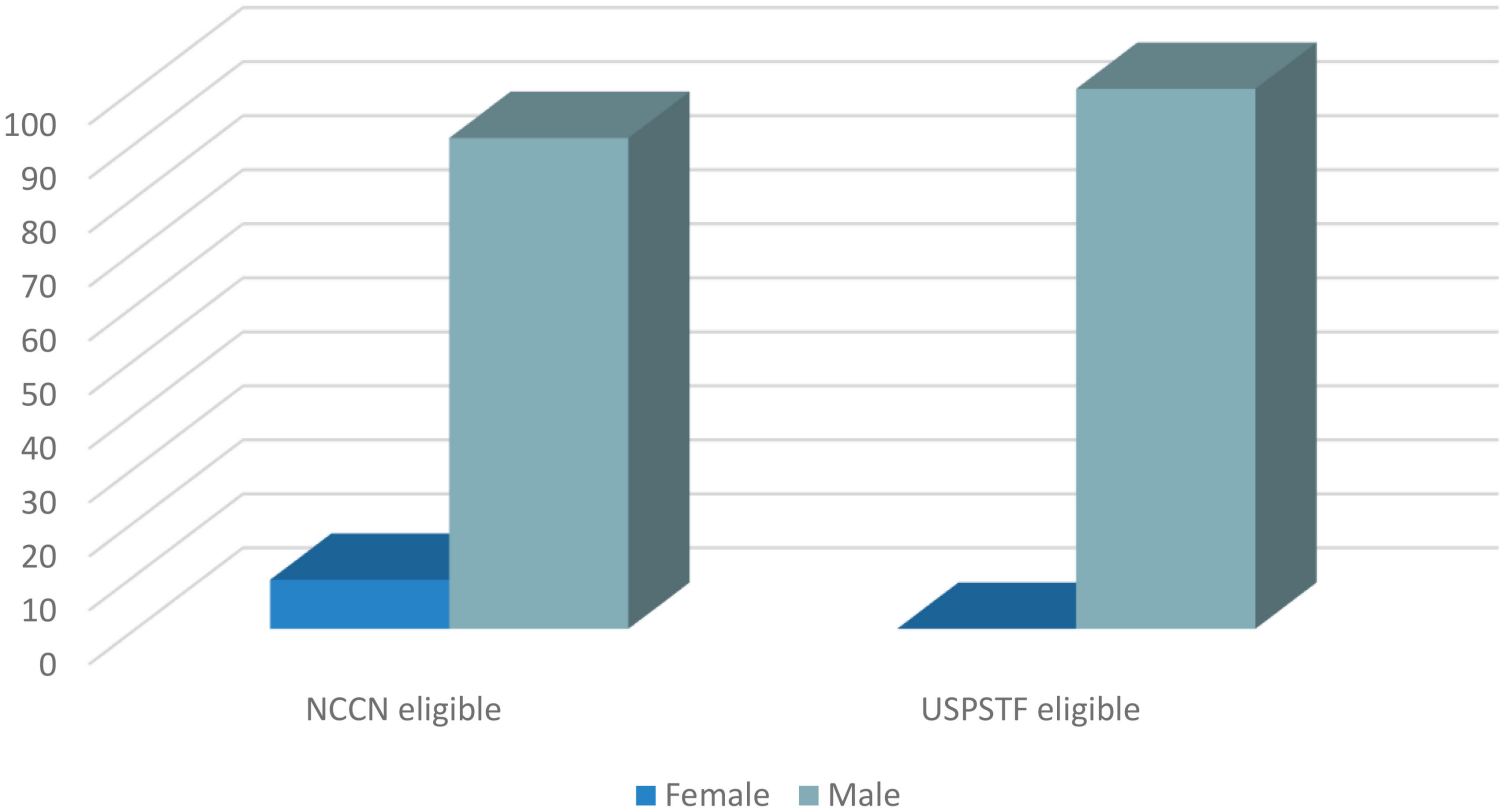
Sex of eligible subjects that underwent LDCT.

**Figure 2:**
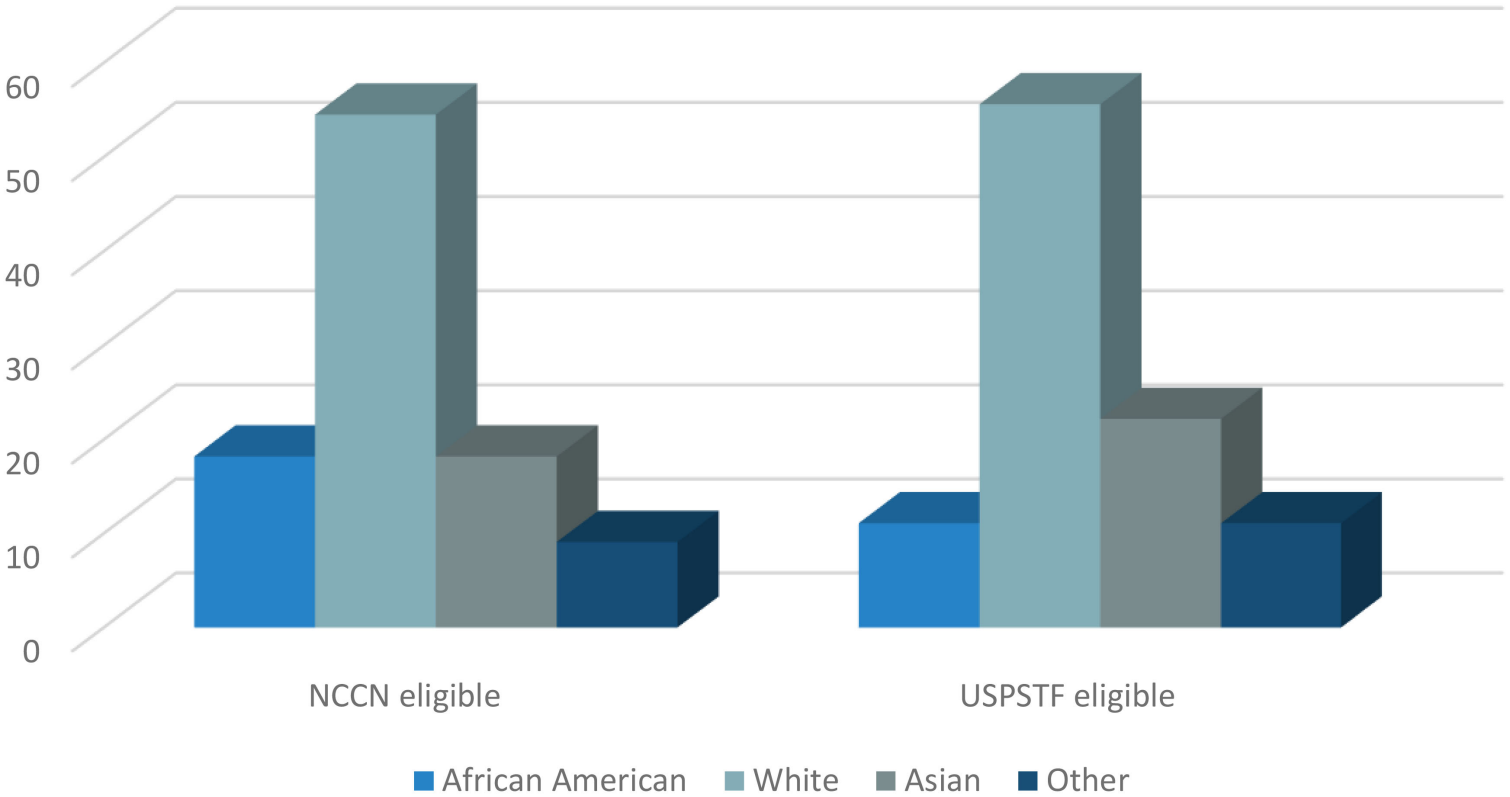
Race of eligible subjects that underwent LDCT.

**Figure 3:**
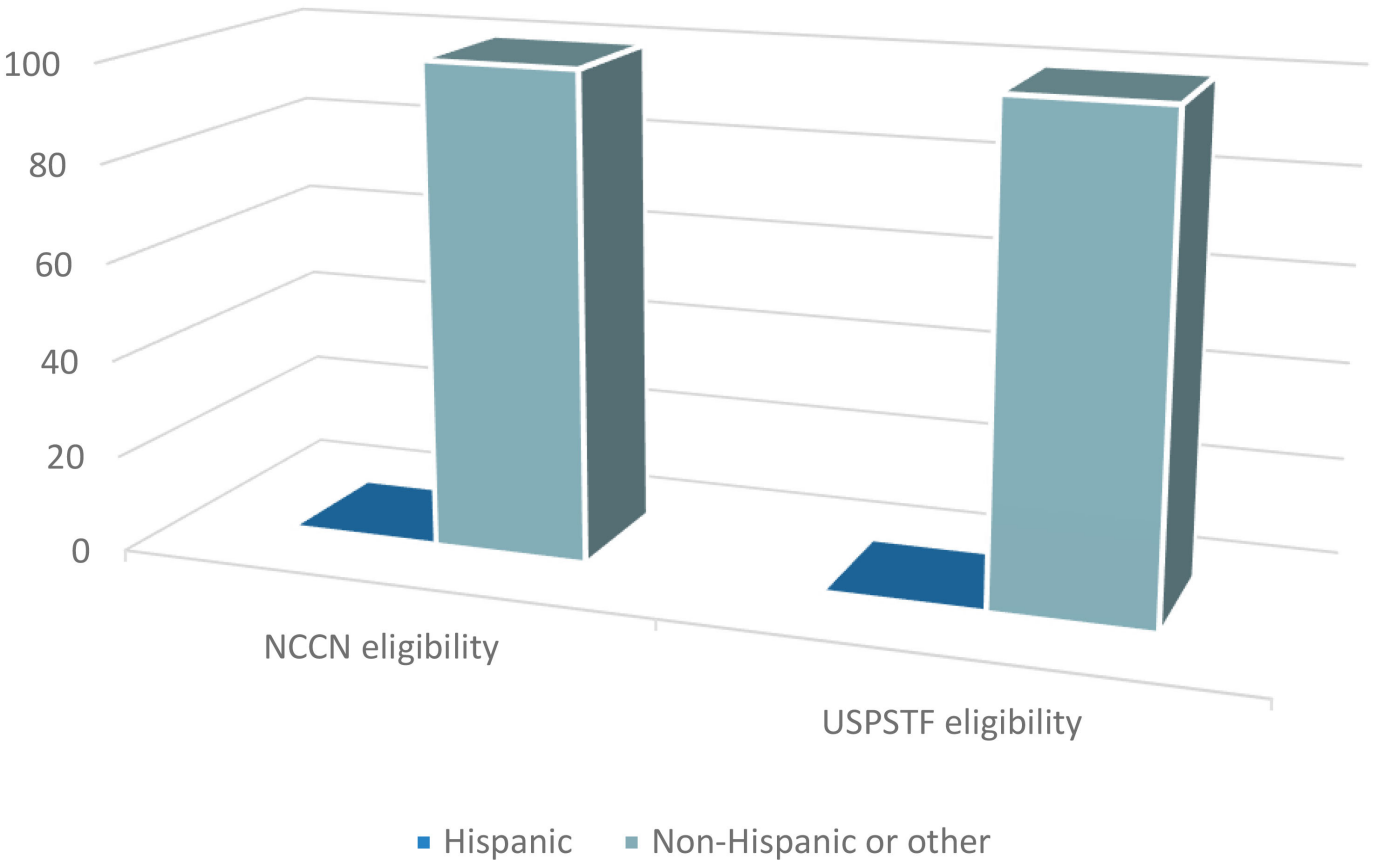
Ethnicity of eligible subjects that underwent LDCT.

As published in our original study (13), only 4.0% and 4.8% of the subjects that fulfilled NCCN and USPSTF eligibility criteria, respectively, underwent LDCT (95% exact CI: 2.0, 7.0 and 2.2, 9.0). Ninety one percent of the subjects that had LDCT uptake in the NCCN eligible group were men (**Figure 1**). Similarly, 100% of the subjects that underwent screening in the USPSTF eligible group were men (**Figure 1**). Of the NCCN eligible individuals that underwent screening, 54.5% self-identified as White, 18.2% as African American, 18.2% as Asian, and 9.1% as other (**Figure 2**). None of the subjects that had LDCT in this group self-identified their ethnicity as Hispanic (**Figure 3**). Comparably, in the USPSTF eligible group that underwent LDCT, 55.6% of the subjects self-identified as White, whereas 11.1% identified as African American, 22.2% as Asian, and 11.1% other (**Figure 2**). No subjects self-identified as Hispanic (**Figure 3**). An association between screening and age, gender, race, and ethnicity could not be evaluated due to the low sample size of individuals that underwent LDCT screening.

For additional information regarding screening according to smoking status and staging at diagnosis, please refer to original study (13).

## Discussion

Vast literature exists to support the disparities in lung cancer screening eligibility and implementation in the African American population (11, 14, 15). However, to our knowledge, our study is the first one to evaluate the rates of screening eligibility according to race and ethnicity amongst the different criteria, set forth by the NCCN11 and USPSTF13. Contrary to other studies which analyzed subjects eligible for screening, our cohort of patients was individuals with lung cancer that were retrospectively assessed for NCCN11 and USPSTF13 lung cancer screening eligibility and LDCT uptake prior to their diagnosis of lung cancer. We found significantly lower rates of USPSTF13 eligibility for Hispanics compared to non-Hispanic or others. Our study suggested that the differences in the screening criteria between NCCN11 and USPSTF13 influenced eligibility amongst Hispanics, who were noted to have higher tendencies to fulfill NCCN11 than USPSTF13 criteria.

Evidence has suggested that underrepresented groups tend to have a higher risk of LC at a younger age and with less smoking exposure (16). Findings such as these provided the impetus for the latest changes in the screening guidelines, the most important of which decreased the age and pack-year requirements. In March of 2021, the USPSTF updated their lung cancer screening recommendations to include adults aged 50 (formerly 55) to 80 years who have a ≥20 (formerly 30) pack-year smoking history and currently smoke or have quit within the past 15 years (17). The 2020 guidelines set forth by the NCCN identify high-risk individuals as those aged 55-77 (formerly 74) with a ≥30 pack-year history of smoking who are current smokers or have quit within 15 years, or age ≥50 with a ≥20 pack-year history and one additional risk factor (9). In a recent study which retrospectively observed these changes in eligibility under the updated USPSTF guidelines, the proportion eligible for screening among current and former smokers increased by 76.7% for African American and 78.1% for Hispanic populations. However, compared with white individuals, African American and Hispanic individuals still had lower odds of eligibility (18). Clearly, even with updated guidelines, disparities exist in LC screening among underrepresented populations. Another cross-sectional retrospective survey study evaluated the association between race and ethnicity and lung cancer screening eligibility in subjects from 20 states. The rates for screening eligibility increased from 12%, 4%, and 7% to 15%, 5%, and 9% in White, Hispanic, and African Americans, respectively, under the new screening guidelines. Nevertheless, African American (p<0.001) and Hispanic (p<0.001) respondents were still less likely to fulfill lung cancer screening eligibility than Whites. Additionally, the study found no statistical association in the rates of screening eligibility for racial and ethnic minorities under the revised USPSTF21 guidelines (p=0.76) (19). Lastly, one study evaluated whether the updated USPSTF21 lung cancer screening recommendations would ameliorate racial disparities in screening eligibility. It found that although the revised guidelines increased the eligibility of minorities compared to the USPSTF13 guidelines, racial and ethnic disparities may inadvertently increase (20).

While this study primarily focused on screening eligibility of Hispanic patients, a retrospective objective analysis of the data from our previous study revealed potential disparities in screening implementation and LDCT uptake according to sex, race, and ethnicity. Despite 52% and 20% of the self-identified Hispanic subjects with lung cancer in our study being eligible under NCCN and USPSTF criteria, respectively, none underwent screening prior to their diagnosis of lung cancer. Additionally, White and male subjects had higher rates of screening uptake. While, liberalizing and loosening the lung cancer screening guidelines is the first step to increase the rates of screening eligibility for high-risk individuals, it comes at the risk of perpetuating pre-existing disparities that exist for underrepresented groups. Disparities in screening will continue to widen if factors including sex, race, and ethnicity are not taken into consideration. Additionally, assessing and weighing the individuals’ social determinants of health such as socioeconomic status, living environment, and health insurance to understand the risk of lung cancer and ability to undergo LDCT is of paramount importance to increase the rates of lung cancer screening and early diagnosis for all groups.

Despite sharing some common characteristics, one size does not fit all when it comes to sex, race, and ethnicities - and we must take into account the different risk factors and characteristics each group possesses in order to seek health equity for our most vulnerable populations. Possible ways to mitigate these disparities include making active efforts to improve inclusivity in clinical trials and continuing to revise and expand the inclusion criteria for LDCT, incorporating social determinants of health Ongoing prospective studies to understand how these factors affect the risk of lung cancer and create guidelines to integrate these will be of need to optimally close the gap in lung cancer screening disparities.

## Data availability statement

The original contributions presented in the study are included in the article/supplementary material. Further inquiries can be directed to the corresponding author.

## Author contributions

ME assisted in data collection and the writing of the manuscript. NS overlooked the study and assisted with manuscript proofread. All authors contributed to the article and approved the submitted version.

## Conflict of interest

The authors declare that the research was conducted in the absence of any commercial or financial relationships that could be construed as a potential conflict of interest.

## Publisher's note

All claims expressed in this article are solely those of the authors and do not necessarily represent those of their affiliated organizations, or those of the publisher, the editors and the reviewers. Any product that may be evaluated in this article, or claim that may be made by its manufacturer, is not guaranteed or endorsed by the publisher.
